# Improving the Engagement of Underrepresented People in Health Research Through Equity-Centered Design Thinking: Qualitative Study and Process Evaluation for the Development of the Grounding Health Research in Design Toolkit

**DOI:** 10.2196/43101

**Published:** 2023-02-28

**Authors:** Alessandra N Bazzano, Lesley-Ann Noel, Tejal Patel, C Chantel Dominique, Catherine Haywood, Shenitta Moore, Andrea Mantsios, Patricia A Davis

**Affiliations:** 1 Department of Social, Behavioral, and Population Sciences Tulane University School of Public Health and Tropical Medicine New Orleans, LA United States; 2 College of Design North Carolina State University Raleigh, NC United States; 3 Global Impact Board New Orleans, LA United States; 4 Louisiana Community Health Outreach Network New Orleans, LA United States; 5 Louisiana State University Health Sciences Center New Orleans, LA United States; 6 Public Health Innovation & Action New York, NY United States

**Keywords:** health equity, patient participation, health behavior, universal design, human-centered design, COVID-19

## Abstract

**Background:**

Health inequalities are rooted in historically unjust differences in economic opportunities, environment, access to health care services, and other social determinants. Owing to these health inequalities, the COVID-19 pandemic has disproportionately affected underserved populations, notably people of color, incarcerated and formerly incarcerated individuals, and those unable to physically distance themselves from others. However, people most strongly impacted by health disparities, and the pandemic, are not frequently engaged in research, either as researchers or as participants, resulting in slow progress toward improving health equity. Establishing ways to foster the engagement of historically excluded people is crucial to improving health equity through patient-centered health research.

**Objective:**

This study aimed to assess the use of equity-centered design thinking (EDT) for engaging community members in research prioritization related to COVID-19. The co-design methods and subsequent production of a toolkit that can be used for engagement were assessed through process evaluation and qualitative methods.

**Methods:**

Process evaluation and qualitative inquiry, using reflexive thematic analysis, were undertaken to examine the use of EDT. Patient community members and stakeholders remotely partnered with design and health researchers in a year-long digital process to cocreate capacity-building tools for setting agenda for research regarding the impact of COVID-19 on health outcomes. Through a series of 3 workshops, 5 community partners engaged in EDT activities to identify critical challenges for the health and well-being of their communities. The subsequent tools were tested with 10 health researchers who provided critical input over the course of 2 workshops. Interviews with co-designers, project materials, and feedback sessions were used in the process evaluation and finalization of an equity-centered toolkit for community engagement in research. Data from the co-design process, meetings, workshops, and interviews were analyzed using reflexive thematic analysis to identify salient themes.

**Results:**

Process evaluation illustrated how the EDT co-design process offered an approach to engage patient partners and community stakeholders in health-related research around COVID-19. The participants expressed satisfaction with design thinking approaches, including creative activities and iterative co-design, as a means of working together. Thematic analysis identified 3 key themes: the value of authentic partnerships, building trust and empathy through design, and fostering candid dialogue around health and social issues impacting historically underrepresented and underinvested communities.

**Conclusions:**

The project addressed the need to test EDT strategies for fostering inclusive community engagement in health research agenda setting and provided an alternative to traditional top-down models. Despite the increasing use of human-centered design in health, few projects explicitly include equity in design thinking approaches. The use of methods and tools to intentionally engage underrepresented stakeholders in the process of research agenda setting and equitably sharing power between researchers and community members may improve health research, ultimately improving health equity.

## Introduction

### Background

Health inequalities are rooted in historically unjust differences in economic opportunities, environment, access to health care services, and other social determinants. In particular, structural racism continues to be a key determinant of health in the United States [1]. Owing to these health inequalities, the COVID-19 pandemic has disproportionately affected underserved populations, notably people of color, incarcerated and formerly incarcerated individuals, and those unable to exercise physical distancing [2]. Populations that were strongly impacted by COVID-19, such as Black Americans with chronic health conditions [3], have also been historically excluded or underrepresented in health research, both as participants and researchers [4]. Unless participants from diverse backgrounds are equitably involved in research that centers on individuals with lived experiences, an important opportunity to reduce health disparities will continue to be missed. While recognizing how racism and historical injustices, in all of their manifestations, have a deep relationship with current health inequities, the potential to reshape institutional frameworks in ways that promote rather than diminish equity is available through redesigning systems.

Patient-centered outcomes research (PCOR) and comparative effectiveness research (CER) are important approaches for identifying ways to prevent and mitigate ill health, where research priorities and agendas originate from authentic patient needs and preferences [5]. Patients, defined as individuals who have lived experience with an illness or injury [6], are infrequently consulted when researchers develop funding proposals or delineate research questions, which are more often arrived at in a top-down fashion from funding agencies and biomedical research organizations. PCOR helps patients and their caregivers communicate and make informed health care decisions, allowing their voices to be heard in assessment of the value of health care options [7]. CER compares the effectiveness of ≥2 interventions or approaches to health care, examining their risks and benefits, and can be used specifically to explore interventions for reducing or eliminating disparities in health and health care [8]. The COVID-19 pandemic has provided an opportunity to amplify the voices of disproportionally impacted and underrepresented communities and promote equity in health research. Previous work on the engagement of underrepresented groups in research has focused on traditional methods of community-based collaboration, such as consultation or advisory boards [9]. However, the kind of solutions urgently needed to combat an unprecedented pandemic and to redesign health for improved equity requires innovative approaches that go beyond traditional problem-solving public health methods [10].

### Prior Work

Design thinking, or human-centered design, is an approach that draws upon a designer’s toolkit to put people at the heart of understanding, experimenting, and acting to address challenges. It uses a constructive and experiential mindset rooted in the needs and context of the end users of a product or service to develop novel solutions [11]. It is grounded in empathy for the needs of a community, gaining a clearer understanding of the problem through direct engagement, and developing solutions *with* rather than *for* people who will use them [12]. It can be seen as collectively revolving around several core concepts, including empathy for users, a discipline of prototyping to gain insights, and tolerance for ambiguity, in which failure is seen as a necessary part of learning through iteration [13].

In the context of the early part of the COVID-19 pandemic, in which evidence evolved rapidly, necessitating adaptation for behavior change, a potential bridge was created between design and public health, where design mindsets, skills, and processes could be usefully applied to prioritizing research on COVID-19, creating a unique opportunity for the intersection of these 2 disciplines. A design approach lends itself to PCOR and improving patient experience, as it is human-centered, multifaceted, and may be used in several ways through remote or in-person techniques.

An important element that must be included in design for social good, also known as liberatory or emancipatory design [14], is an equity-centered design thinking (EDT). The theoretical focus of this approach on the power structures that shape accessibility and inclusion fosters the equitable participation of minoritized populations. EDT can be described as a way of approaching problem solving by acknowledging and using the roles of people, systems, and power [15]. In this practice, minoritized communities are purposefully involved as co-designers, with the goal of developing direct solutions to relevant social issues and community needs [16]. EDT may be used with groups that are marginalized for diverse reasons, such as race, ethnicity, gender, and sexual orientation. Although efforts have been made to highlight the importance of equity and engagement in improving health in various fields, such as aging [17] and reproductive justice [18], there remains a gap in the literature around research using equity-focused approaches in design thinking or human-centered design.

In this study, we used process evaluation and qualitative data analysis to assess a project aimed at building the capacity of community members from underrepresented populations to engage in research prioritization related to the impacts of COVID-19 among communities through an innovative, equity-centered design approach. This study evaluated the use of co-design strategies to build designerly capabilities and mindsets to influence the agenda setting for PCOR and CER related to COVID-19. Involving underrepresented people in research through equitable engagement is key to improving health equity, particularly where health disparities have resulted from social injustice and structural racism.

## Methods

### Setting and Participants

Louisiana was a hot spot in the early stages of the pandemic [19], and New Orleans residents were among those most affected by the transmission of COVID-19 in the country, with limited access to preventive care and treatment. New Orleans is a majority Black city [20] with unique health challenges relative to other parts of the country (eg, higher rates of hypertension, diabetes, cardiovascular disease, and obesity) [21]. Given the history of disasters in New Orleans, including Hurricane Katrina; high levels of chronic disease comorbidity; and prolonged economic challenges, the impact of COVID-19 has been compounded and often less visible, such as in the case of trauma and mental health needs.

Potential co-designer participants were identified and recruited through community-based networks using purposive sampling. The recruitment strategy required community members from populations underrepresented in health research on COVID-19, with lived experience and insight into local community needs related to the pandemic, who could commit to a 1-year project. A total of 5 adult participants joined the project all of whom self-identified as Black or African American and from the New Orleans community including 2 local leaders active in neighborhood or community organizing, 1 formerly incarcerated person, and 2 participants involved in health or care services for local communities. There were no age or gender requirements for inclusion, yet the group was varied and not homogenous with respect to those characteristics (to protect privacy, these have not been included here). Furthermore, 2 community participants contracted COVID-19 during the course of the project and recovered. The team of researchers and staff carrying out the project prioritized awareness of positionality and power dynamics in implementation; they self-identified as a White public health researcher from the city, a Black (non-American) designer and design researcher not from the city, a Black community health research coordinator from the city, and a South Asian research coordinator and doctoral student in public health who was not from the city. A total of 10 health researchers participated in workshops at the later stage of the project to test draft materials developed through co-design and were purposively recruited from patient centered research networks supported by the funding institute.

The methodology used in this study is presented in Table 1, which outlines the co-design, process evaluation, and thematic analysis.

**Table 1:** Methodology.

Approach	Methods and participants	Research objective	Reference
Co-design	EDT^a^ workshops with community members and health researchers	To improve engagement in health research through the development of a toolkit	Emancipatory design and liberatory design
Process evaluation	Analysis of the interviews with workshop participants Analysis of the workshop materials and minutes of research team meetings Descriptive analysis of workshop co-design processes including participants of the study research team and workshop participants	To assess the EDT co-design process that resulted in the development of a toolkit	Developmental evaluation
Qualitative analysis	In-depth, debriefing interviews with workshop participants Minutes of study research meetings Analysis of transcripts from all workshops	Thematic analysis of text to identify salient themes	Reflexive thematic analysis

^a^EDT: equity-centered design thinking.

### Co-design Methods

#### Overview

A series of videoconference convenings were held in New Orleans with 5 community members between September 2020 and July 2021, namely a first meeting, 3 design workshops, and a closing meeting. All convenings and workshops were held remotely via Zoom (Zoom Video Communications, Inc) owing to the COVID-19 pandemic, and each lasted for approximately 90 minutes. The research process focused on connection and relationship building to increase the capacity for engagement among the stakeholders and research team, as well as iterative developmental evaluation to ensure responsiveness to partners’ needs and innovation.

First, an introductory meeting was convened with the stakeholders, which was facilitated using the VISIONS model (VISIONS, Inc) of multicultural communication [22,23], to set the stage for equity-centered work. The VISIONS model of addressing diversity, equity, and inclusion was identified as highly pertinent given that it was developed in 1984 by 3 Black women who grew up in legalized segregation and a White Jewish man who grew up during the civil rights movement to answer the question, “How do we include people who have been historically excluded from white, mostly male institutions?” The research team worked with 2 local community-based facilitators from VISIONS in advance to discuss how researchers were approaching the work and some of the nuances of building equitable partnerships with community members. They then cofacilitated the first stakeholder meeting, incorporating a variety of approaches and techniques to address working together across differences and what people think, what they do, and how they feel. The communication framework was emphasized to begin establishing trust and comfort for open dialogue and communication throughout the project and to ensure that equity and inclusion were at the heart of the project. Stakeholders were also introduced to basic design thinking concepts in the first meeting, and they completed a practice design thinking activity. During the initial gathering, Mural (Mural), a web-based whiteboard, was introduced as a tool to engage stakeholders and facilitate communication among them in the remote context of the workshop. The participants were guided in practice using the features and tools of Mural and in getting accustomed to using technology together as a group.

Over the course of the next several months, 3 workshops were conducted to co-design an agenda-setting process for research prioritization with patient stakeholders and to foster engagement among the stakeholder groups for research on COVID-19. The workshops included both topic nomination, the identification of major themes of concern, and a prioritization process to rank health topics in the order of their importance for research. The co-design methods that were used included visual ethnography or photo sharing; future thinking around health and well-being (eg, coming up with utopian and dystopian health headlines from the future); cultural inquiry to understand patients’ daily routines and experiences with illness and health; and prototyping, that is building things with one’s hands to encourage creative thinking and stimulate new ideas. Table 2 illustrates the meeting events and topics.

**Table 2:** Stakeholder engagements during the Grounding Health Research in Design process.

Meeting	Topic	Illustrative content
1	Getting to know each other and forming agreements on how to work together	Communication framework established Introduction to web-based whiteboard technology
2	An introduction to visual ethnography	Visual narratives about the cultural and health impacts of COVID-19
3	Rapid Critical Utopian Action Research	Dream building for future health Lessons learned from the pandemic
4	Low-fi approaches for engagement	Working with hands and commonly found materials to spur creativity and storytelling
5	Closing convening and sharing	Reflection on the work done together Next steps

#### Workshop 1: Visual Ethnography

All the workshops began with warm-up activities, through which group members got to practice creativity while also getting to know each other, hear each other’s stories and experiences, and build stakeholder-researcher trust. When prompted to share what New Orleans looked like to them, the stakeholders began relating both shared and individual experiences of a common culture and cuisine (eg, gumbo) and things they loved about their city (eg, “waving to my neighbors in the evening”). This activity fostered a sense of social cohesion based on the group members’ pride in their common place and culture and underscored the importance of such activities in building stakeholder-researcher trust and comfort for sharing potentially sensitive information with a new group of people.

In the first workshop, visual ethnography was explored, where stakeholders and researchers shared photos related to their experiences during the COVID-19 pandemic. Mural, a web-based whiteboard, was used to organize people’s experiences into themes as a group, and these themes were then voted upon to create priorities for research.

#### Workshop 2: Rapid Critical Utopian Action Research

During the second workshop, an adapted version of Critical Utopian Action Research [23] was used to enhance future thinking. The stakeholders were asked to imagine that they were in the year 2030, when the pandemic was over, and to consider the advice they would give people in the past. First, they were asked to think about what they would tell people in 2019 before the pandemic to help them prepare better. In the first exercise, titled “Coulda, woulda, shoulda,” the participants imagined what they could, should, or would have done to prepare for the pandemic. In addition, they used voting to prioritize health research topics, particularly mental health.

#### Workshop 3: Cultural Inquiry

The approach for the third workshop was cultural inquiry and was based on an applied anthropology method used by interaction design researchers known as cultural probes [24]. Before the workshop, the stakeholders were mailed the materials to be used during the session. To build anticipation and camaraderie in the web-based environment, the stakeholders were instructed not to open the packet of materials until the workshop meeting. The low-technology materials and methods drew on studio art pedagogies and descriptive storytelling while also allowing the assessment and comparison of participants’ responses to the digital technology–centered methods using the web-based whiteboard in the previous workshops. Activities in this workshop included the completion of “A COVID story,” similar to Mad Libs; a fill-in-the-blanks word game; and the creation of a prototype of something that could help with mental health during a pandemic using basic craft supplies and materials.

The engagement of project researchers and staff as participants in all activities alongside patient stakeholders was important to the EDT strategy. In addition, all the workshops placed a strong emphasis on warm-up activities to build stakeholder-researcher trust, foster creativity, and increase comfort for engaging in the design thinking process.

After the 3 workshops, a final convening was held with the community members to discuss the stakeholders’ overall experiences participating in the project and gather their feedback on the piloted EDT activities and inputs on the development of a toolkit intended for researcher stakeholders interested in adopting EDT within their own communities and organizations.

### Process Evaluation of Co-design: Data Collection and Analysis

The study used process evaluation, based on Patton’s [24] model of developmental evaluation, which refers to “long-term, partnering relationships between evaluators and those engaged in innovative initiatives and development. Developmental evaluation processes include asking evaluative questions and gathering information to provide feedback and support developmental decision-making and course corrections along the emergent path. The evaluator is part of a team whose members collaborate to conceptualize, design and test new approaches in a long-term, ongoing process of continuous improvement, adaptation, and intentional change.”

Data for the process evaluation of the co-design and engagement were collected through stakeholder interviews with partners and researcher usability workshops held with potential users of the developed EDT toolkit. Supplementary data were accessed through the text review of internal program documents from workshops and convenings, which cataloged participant exercises, and the text and visuals created in the workshops through templates using web-based whiteboards, screenshots, digital photography, video, and audio. In-depth debriefing interviews were held with stakeholders after each workshop to assess their experiences participating in the specific EDT activities piloted as well as their thoughts on how useful these would be for working with their communities.

The research team also held weekly check-in meetings to process their own experiences participating in the project as well as reflect on what they thought went well and did not go well in the stakeholder workshops, practicing iterative development for responsiveness in evaluation. They reviewed notes, materials, and transcripts from the workshops to determine what would be included in the toolkit and to guide and inform the process of refining future workshop content and delivery.

### Thematic Analysis

On the basis of Braun and Clarke’s [25] model of reflexive thematic analysis, data were assessed for salient themes. The reflexive approach to thematic analysis pays attention to the active role that researchers play in the creation of knowledge [26]. The thematic analysis process included familiarization, generating initial codes, searching for themes, reviewing themes, and defining and naming themes. The material used for analysis is shown in Table 1, and after the analysis, illustrative quotes were selected for presentation to elucidate the link between the text and identified themes. The Consolidated Criteria for Reporting Qualitative Studies (COREQ) 32-item checklist was used to ensure transparency and quality throughout [27].

### Ethics Approval

The study was reviewed and approved by the Tulane University Institutional Review Board (approval number 2020-1369). All the participants provided verbal informed consent. The study data were anonymized to protect privacy, and confidentiality was maintained through the use of encrypted computers and files and the removal of personal identifiers from study documents. The participants were compensated for their time in accordance with the funding body’s standards for patient participation in engagement research.

## Results

Findings from the study are presented in two sections: (1) results of the co-design process evaluation across workshops with a description of activities and quotes illustrating the experiences of participants and (2) thematic analysis with a description of and quotes illustrating the themes.

### Process Evaluation of Workshops

Community stakeholders expressed positive sentiments around their experiences participating in co-design and candidly discussed the content covered in the workshops in relation to their communities and the organizations where they work to address key issues. These issues included relying on integrated community networks and relationships, using digital platforms for communication, and leveraging increased interests in health and well-being to build community awareness around health outcomes. Several stakeholders felt that using co-design, specifically EDT activities, within their communities could improve health care, social services, and employment engagement. However, some noted challenges such as ensuring that the activities meet the needs of people with fewer resources or those who may not have time to participate in lengthy activities because of work and family responsibilities as well as concerns about trust and privacy. Specific examples of activities and experiences are given in the subsequent paragraphs.

The first workshop, with its photo sharing activity, allowed for all participants, including the researchers themselves, to connect over family and common experiences. This activity was considered particularly impactful for fostering equitable partnerships, and it was discussed during the workshop as well as in follow-up interviews:

Dealing with these people [researchers] you get to know them as a real person, you get to know them not just a professor or...this lady who runs a research program. I got a chance to feel a part of her, get a real sense of her and that just brought on a different [way]...how you feel about participating in the workshop and how successful you want it to be because you know, people are being real.

Community Partner 1

During the second workshop on Rapid Critical Utopian Action Research activities, the participants were exposed to future thinking, which shifted their perspective to “dreambuild” a positive future. They thought of the advice they would give, such as urging the people in 2019 to see their primary care physician, considering how interrupted routine medical care would become owing to the pandemic, and to consider ways to protect their mental health. One of the participants focused on the financial stress the pandemic placed on the members of her community:

I should’ve educated my community on financial freedom because I realized during the pandemic that that was the biggest part of everybody trying to be safe was being concerned about their finances.

Community Partner 3

In another future-focused activity, the participants imagined utopian and dystopian news headlines about health in the future. Through this activity, they reflected on some of their key takeaways from living through the COVID-19 pandemic and the introduction of the COVID-19 vaccine. A headline proposed by one of the participants is “Due to the research for the COVID vaccines, other safe and effective vaccines were made more quickly in the future.” Another participant proposed the following headline: “African Americans’ disbelief in the vaccine because of past history and the fear of being used as guinea pigs caused more death!” Furthermore, yet another participant proposed the headline “Americans no longer believe in the health care system.” Issues of trust and lack of, or lost, trust in health care emerged in most future headlines framed during this activity.

Through an analysis of workshop notes and transcripts after the first 2 workshops and as part of the iterative design process, the research team sought to reduce technological challenges. The third workshop adopted a lower-technology approach using paper materials and prototyping with hands, rather than an approach involving heavy use of a computer.

The “Mad Libs”-style activity in the third workshop titled “A COVID story” allowed the stakeholders to share stories of their friends and loved ones that tapped into community perceptions and norms. One of the stakeholders crafted the following story:

My story is about Michelle...The biggest issue faced related to coronavirus is being separated from her family. She lost her father, and then she just lost her aunt within the past month from COVID-19. Her family is really going through a tough time with not being able to see her grandmother...having to minimize the funerals to just a few people was really devastating to their family. They just felt a lot of strain on their family. They feel like they should’ve been provided better services and instructions from the government.

Community Partner 1

The participants were particularly responsive to the story telling activity, with several noting that it would be an effective way to obtain feedback from members of their communities. Others echoed the sentiment that personal stories about oneself or someone in one’s life are always effective at getting people to speak more comfortably, particularly about sensitive topics.

Eco-mapping, the activity in which the participants identified the resources that were around them and their families, spurred dialogue around the distrust of health care professionals and researchers. For the prototyping activity, a mainstay of design thinking approaches, the participants were provided a package of basic materials (eg, colored paper, sharpie marker, sticky notes, pipe cleaners, and paper plates) and were asked to create something that could help with mental health during the pandemic. Creations included a bunker filled with food and medical supplies, a pod-finder phone app to locate nearby individuals to isolate with, a shrine to commemorate loved ones lost to COVID-19, and a time travel device to transport people to their happiest childhood memories. The participants described prototyping as follows:

I didn’t think I was really artsy until I started doing these kinds of projects...I was like I’m not going to be able to do this, you guys, and they were like ‘Ok we’ve got 8 minutes and we got all this stuff right here’ and I’m like ‘This is stuff that children play with, I don’t know what to do with pipe cleaners,’ and you sit there and you see your imagination runs so wild. Like just in this moment, I thought of my god, this will be so great, a great idea to have this bunker underground...

Community Partner 1

However, one of the participants expressed being unsure what to do with the materials:

I don't know anything to do. Looking at this was like me looking at Latin. It was just like, I don't know what to do with this.

Community Partner 2

After the workshops, one of the stakeholders reported that she had already spoken with the board of her community organization about design thinking and wanting to incorporate it into their work. Another individual described how she shared her knowledge of and interest in design thinking with friends and peers with whom she worked in her community:

I always explain this to people, we were always taught to think inside the box, growing up in a society where your process was supposed to be the same—you learn your ABCs and your 123s and that was the way we were taught to live...now in design thinking you don’t have to think in that process if you don’t want to, it’s not the only process that’s the right process and that’s what design thinking is. It’s thinking outside the box. So whenever someone says ‘what made you think of that?’ I say ‘design thinking made me think of that! I just thought about how it’s always been done and I think about how we live today, and how can we enhance what’s always been done to how we can do it today...I was just thinking outside of the box, that’s all.’

Community Partner 1

Finally, the participants expressed that the EDT processes used for creating the toolkit could have value beyond the study for their own communities, for connectedness, and for the broader promotion of health equity. One of the community participants stated the following:

I know the ultimate goal from this is to take what we’re bringing to this to the communities in some type of way, I’m very excited to see how that is actually going to come forth. The things we are discussing are actively the things people in our community are talking about behind closed doors anyway so if we could bring it to the actual community in some kind of way to have this kind of connectedness in the time of COVID where we can’t really be connected, I think that could be amazing at this time and bring about a connectedness in communities that we don’t really have...

Community Partner 3

Table 3 presents the information derived from the process evaluation of stakeholder experiences with the co-design activities and the reported contribution they made to the equity-centered design process.

**Table 3:** Activities and equity-centered approaches.

Activity	Description	Equity-centered outcomes
Warm-ups	Participants were asked to describe what *their* New Orleans looked like	Fostered bonding over shared culture and cuisine, which created a sense of group cohesion Trust and empathy through place and culture (contributions to the equity-centered design process)
Photo sharing	Participants shared photos of how the pandemic affected their behaviors	Facilitated nonhierarchical conversations across and among stakeholders from different backgrounds (contribution to the cocreation of research)
Future headlines	Participants created news headlines from the future	Prompted dialogue around issues of trust and lack of or lost trust in health care providers, institutions, etc (health topic prioritization)
“Coulda, woulda, shoulda...”	Participants imagined that they were in the future and thought about what they would tell people in 2019 before the pandemic to help them prepare better	Revealed social stressors related to the pandemic (health topic prioritization)
Design Libs: a COVID story	Participants completed a fill-in-the-blank COVID-19 story, similar to Mad Libs	Allowed group members to share stories about COVID-19 through the voice of someone else, revealing the social, environmental, behavioral factors associated with this health topic; and the experiences and stories of their family and friends (health topic prioritization process)
Prototyping	Stakeholders were asked to use craft materials to create a prototype of something that could help during the next pandemic	Working with hands and tangible materials supported processing experiences with the COVID-19 pandemic and finding solutions (contribution to the cocreation of research)

### Researcher Workshops

The final step in the toolkit development process involved holding researcher usability and feedback workshops, with the support of partners at the Louisiana Public Health Institute and the patient-centered outcomes research network PCORnet [28]. In an effort to include input from all potential users of the toolkit, the draft was presented to health researchers who could potentially use it in their patient-engaged research through 2 different workshops. These workshops were both didactic in nature, discussing the activities as educators and practitioners, and experiential, such that researchers participated in the activities to obtain a clearer understanding of how to use the methods and to raise any concerns for developmental evaluation. Important questions were raised during these workshops, including how the toolkit activities could be adapted to be used with individuals rather than with a group for patient-engaged research, how to most effectively use the data after completing this process to ensure that the end user can take the research priorities identified forward, and when in the research process the toolkit would be most helpful.

### Cocreation of the Grounding Health Research in Design Toolkit

On the basis of the insights from all participants and reflections on the design activities piloted, the research team and community partners used the information from developmental evaluation to cocreate the Grounding Health Research in Design (GRID) Toolkit for use by health researchers and communities interested in adopting EDT. Stakeholders’ inputs on the utility of EDT activities and selections of activities that would be most impactful for use in community work informed the content included in the toolkit. The team reviewed the toolkit draft resulting from this process and contextualized the evaluation findings to ensure that the toolkit is community-engaged, stakeholder-driven, and adaptable to a variety of topical areas for research prioritization. Throughout the co-design process, specifically while working together to determine activities for inclusion in the toolkit, the participants discussed various equity approaches. The equity-based VISIONS Inc communication framework (presented in the first meeting and used throughout the project) established an environment in which there was room for controversy and discussion.

The final GRID Toolkit includes the most salient activities identified in this study, with examples related to COVID-19. However, it can be used across various health topics and is intended to serve as a resource for improving the engagement of all people. Plain language and simplified text were used throughout the toolkit to improve usability and address questions raised by the community participants. Activities piloted through the workshops, refined through the process evaluation, and that benefited from health researcher feedback were included.

**Figure 1:**
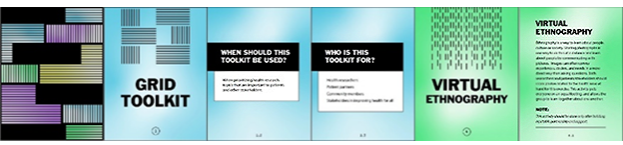
A selection of cards from the toolkit that explains the methods. GRID: Grounding Health Research in Design.

The toolkit may be downloaded in its entirety from the website of the body that funded this research, the Patient Centered Outcome Research Institute [29], and from Louisiana Public Health Institute [30]. It consists of 53 different cards in its present configuration. The objectives of each task are broken down in the accompanying toolkit. It provides background information and detailed, step-by-step instructions for each activity, making it easy for other facilitators to replicate similar activities with stakeholders (Figure 1).

**Figure 2:**
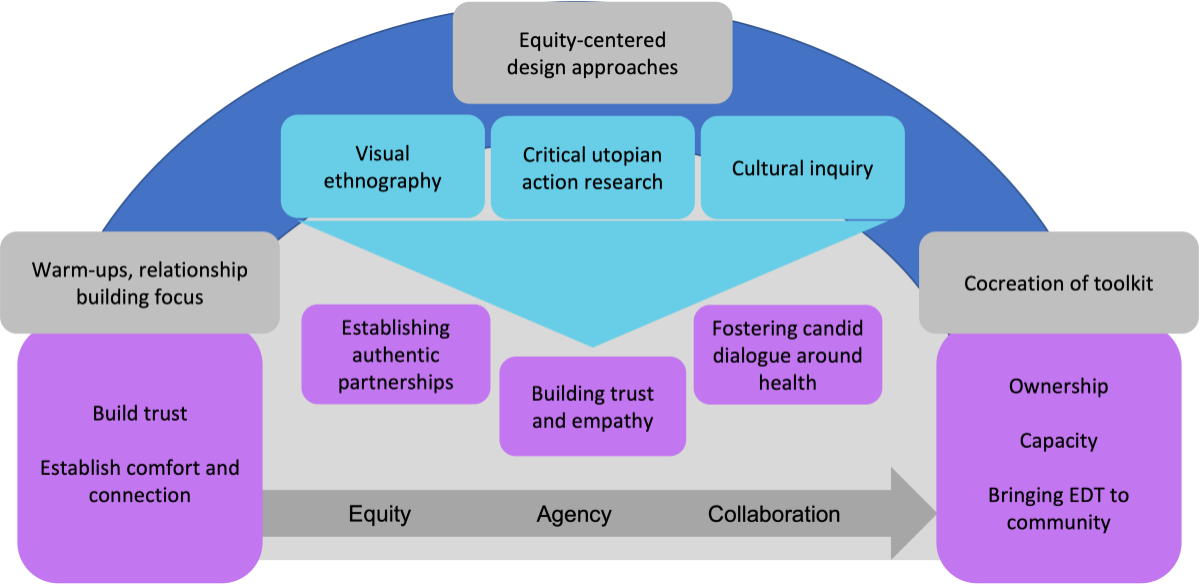
Equity-centered design activities implemented in the co-design process and their outcomes. EDT: equity-centered design thinking.

Figure 2 illustrates the contribution of equity-centered design approaches to fostering equity, agency, and collaboration. The project was initiated with a focus on building relationships, allowing for trust by breaking down hierarchies and establishing comfort through remote partnerships (using technology support). Co-design activities that sought equity, agency, and collaboration were prioritized in the overall process. As shown in the figure, the equity-centered design approaches used in this project facilitated partnerships, sought to build empathy among all team members, and fostered candid dialogue around health and social issues that are not always discussed openly. Cocreation of the toolkit promoted a sense of ownership not only among the researchers but also among the community participants whose opinions and contributions shaped the final toolkit. The capacity to engage in design thinking activities and research prioritization was built in both the community participants and researchers, and the participants strategized how they might bring EDT to their own communities.

### Thematic Analysis

The thematic analysis resulted in the identification of the 3 following salient themes, which are noted in Figure 2 and described in detail in the subsequent paragraphs: the value of establishing authentic partnerships, building trust and empathy, and fostering candid dialogue around health.

Broadly, the thematic analysis of experiences across different categories of participants indicated the theme of the *value of establishing authentic partnerships*, along with the process of building trust and empathy through EDT and how this fostered candid dialogue around health and social issues. Throughout their participation in the workshops and follow-up interviews, both researchers and community participants expressed the importance of engaging in a truly collaborative approach to research. In this context, the community participants vented their frustration with previous involvement in research that felt inauthentic and superficial in nature, as expressed in the following quote:

I’m so tired of researchers who get a grant, get a proposal, put it all together, get halfway through it and then you come to the community because you’re not interacting with the population you need to interact with and here you come, now you need help, right? But you should’ve been there in the beginning and the people should’ve been there from the beginning...And then you get very little information at the end, that’s the other problem, you know, they don’t go back and say this is what we got out of this.

Community Partner 2

Similarly, the health researchers who participated in a usability workshop for the EDT toolkit expressed that they were often less successful than they wanted to be in building authentic and equitable partnerships with community members or patient partners (people impacted by an illness who work together with researchers in health studies). One of the researchers described this as follows:

That’s also something that’s very underestimated within research projects, within budgets, just that capacity, understanding that it takes hours of commitment to build that relationship and to build that trust and comfort with community partners. I think that was huge. We underestimate it...patient partners have been a part of [named health project] for years, but the same issue has been coming up frequently, which really is just them feeling underutilized, and also, they’re just not understanding what the research process is. They're feeling like...the concepts that are being discussed aren’t really tailored to include them in a way that’s meaningful. It’s an ongoing conversation we’ve had for years and so we’ve tried different solutions to address those issues, but it’s an evolving process.

Health Researcher 3

Another researcher spoke of the often-underestimated outcome of relationship building:

We were all talking about how do you measure success and sometimes we’re looking for outcomes, but sometimes the outcome is actually the relationship to be able to continue to do other work

Health Research 2

At the conclusion of the year-long EDT process, the community participants described the level and type of engagement in this project as perspective changing. Being involved in both the crafting of the design process and prioritization of topics was described as changing their perceptions of research and programs intended for their communities:

I would encourage everybody to participate in research workshops to be able to know the steps in how these decisions are made and what effect it has on you and the people that make the decisions like us who participated in the research. I love to know that a real person like myself participated in this study or that study, how they come up to decide that this particular program or process is a good process for me is because someone like myself participated in the process that contained that information.

Community Partner 1

The second theme identified was *building trust and empathy*. The community participants expressed that having the space and ability to discuss health-related community issues in partnership with researchers through EDT allowed for an in-depth exploration to identify key issues and potential solutions. Empathy and trust would also allow for realistic discussions about the needs of the participants.

One of the community participants raised concerns about how researchers, including those using EDT methods, needed to consider the perspectives and lives of those who were not present in the room. Building empathy was described as follows:

From working with communities and neighborhood associations...everybody don’t have a tablet. Everybody just don’t have these things that we’re talking about. We have to think about, we truly have to think about the people who don’t, because we don’t really want to leave them out of certain things. We have to figure out how to include them in research and whatever. Just because they don’t have—they don’t have all the things with them...they might be in their head. We have to figure out how to get this stuff to work for them. That’s all I’m saying.

Community Partner 2

A health researcher who engaged in usability testing of the EDT toolkit spoke of expanding the understanding of community relationships through empathy and trust:

Your comment made me reflect on the context of the community. It’s not just that interaction between the patient or the person that is interacting with the healthcare system. The fact that these people work in the community, they have jobs, they have different relationships with different people in their families, it’s a bigger ecosystem than just that transactional bidirectional relationship of an interview...this helped me think about that.

Health Researcher 1

During one of the workshops with the community participants where everyone shared photographs of ways in which the pandemic changed their life, a research team member shared a photograph of a bag of chips and a glass of wine illustrating the difficulty in eating healthy during the pandemic owing to changes in routine. A community participant later shared in a follow-up interview how the moment when the researcher shared this photo and narrative stuck with her as a moment when she felt more trust and empathy between the researchers and community members:

I loved [researcher’s] photo ‘cause I was going to do something like that and thought I couldn’t. She is one of the leaders of this, and she was really real about who she is and what the pandemic is and how she had to see herself in a new light. The photo just stayed with me and I shared it with my friends to let them know what I encountered [working on this project].

Community Partner 1

The participants conveyed that everyone who was engaged from the start of the EDT process indicated the value placed on their involvement and the subsequent trust and empathy that were built. Other issues of trust were discussed in terms of experiences that the community participants had had with professionals involved in health care, which need to be addressed to build trust and empathy based on engagement and shared experiences:

...we have a lot of trust issues when it comes to healthcare professionals or people involved in the healthcare field. The more we get to know you as a person in my community, the better of a relationship you will have with the people because they get to know that you’re a real person. The fact that you live in a community that you’re servicing, that always makes a big…I want to know if you live around here. Are you breathing the same air I breathe? Your house’s going to flood the same way mine flood or do you drive three hours to come here to my community [and] this person don’t have a clue about what’s going on? It does make a big impact on the people that serves for them to know who you are.

Community Partner 1

The final theme identified was *fostering candid dialogue around health* through the use of EDT activities**,** especially with regard to issues that were exposed by the COVID-19 pandemic. For example, the participants in workshops spoke frankly about how the COVID-19 pandemic put a further strain on the trust of communities, particularly Black communities, in the individuals and institutions delivering health care. The following quotes illustrate the development of this theme:

...they feel like they should’ve been provided better services and instruction from the government. They’re uncertain about whether they want to get a COVID-19 vaccine because, firstly, they don’t trust the healthcare system that’s provided by our government today. Barely anybody in my community relies on healthcare. I got to be honest. Most of them don’t have a primary care doctor. They rely on old-fashioned remedies for healing. Through COVID-19, I think it’s going to get more toward self healing in our Black communities because this has really put a strain on our trust to healthcare. I do believe that it’s going to become more like that for the Black community because this here was a horrible strain on our trust for healthcare from the government. They have mostly gotten their information, like I said, from each other and a few medical providers that they trust along the way.

Community Partner 1

Conversations where health issues were candidly discussed also included dialogue around the skepticism regarding health care providers and medicines:

I’m diagnosed with diabetes, high blood pressure and cholesterol. [After my wife died] I did lose about 20 pounds. I was about 210, I think and I went down to 195 and some. I went for a three or four-month checkup, and then doc say, ‘Oh, all your signs looking good. I1C is lower than what mine is.’ He was saying, ‘Whatever you’ve been doing, keep doing,’ and I said, ‘Okay, I’ll keep not taking that meds. Oh, I ain’t been taking these meds for about three to four months.’ They say once you get diagnosed, it’s hard to get undiagnosed.

Community Partner 4

The tone of equitable interactions fostered an open discussion of health. The participants described communities focusing more on self-healing and the use of traditional practices passed down through families:

I’ve never been in for taking shots, going to the hospital, because like I said, I come from old-fashioned remedies. New Orleans is a place where that has been our lifestyle. I got to be honest, I don’t have a primary care doctor. I don’t. 50 years old, I don’t have a primary care doctor. I pay for services when I desperately need them, and I provide myself with my own medical services, but there are medical professionals I trust. I do have a medical professional that I do trust. Now, I trust him because he has had to deal with the hard time of me.

Community Partner 1

Within one of the community partnered workshops, discussions revolved around how community members obtain COVID-19–related information. One of the participants reflected on frank discussions in her everyday life:

talking about the vaccines in the community and stuff...that made me think about when I go and get my hair done, I always talk about it in there because nobody in there wants to get [the vaccine]. None of the hairdressers are planning to get it, most of the other people who are in there getting their hair done as they’re saying, they won’t either. They asked me a lot of questions, though, because I’ve had both shots at this point. I was thinking about too like where can you or how are we going to get to more people of color to address their concerns and issues with wanting to get the vaccine or not trust in the vaccine and things like that, being that everyone needs to get it, really, the majority of people.

Community Partner 3

## Discussion

### Principal Findings

We have described the cocreation of an equity-focused, human-centered design process to improve the engagement of underrepresented people, particularly Black Americans, in patient-centered health research on COVID-19, with assessment through process evaluation and qualitative thematic analysis. A group of engaged community members worked collaboratively with health researchers to create tools for research agenda setting, which were tested and refined. From the assessment and evaluation of the experiences of stakeholders around the practice and process of co-design and research prioritization, the team incorporated learning to produce a toolkit appropriate to the needs of underrepresented communities for research agenda setting. The tools can be used by communities and health researchers to improve engagement in research prioritization. Key themes identified included the value of establishing authentic partnerships, building trust and empathy, and fostering candid dialogue around health.

In the United States, achieving health equity is highlighted as the primary focus of public health policies and practices [29], and the COVID-19 pandemic placed this in stark relief, as historically minoritized and underinvested communities experienced worse health outcomes in the context of structural racism [30,31]. Although establishing health equity among populations has been an official policy aim in the United States for years, success in eliminating inequities has been difficult to achieve. Finding ways to engage people who have been historically excluded from health research can play an important role in improving patient centeredness in health care research, which is key for eliminating health care disparities and advancing health equity [32]. To enhance health outcomes in general, an important step is to focus on including patients in all aspects of health care—examples of such engagement include shared clinical decision-making and participation in health research topic prioritization [33].

In this study, we presented a process evaluation and thematic analysis assessing the engagement of community partners in health research prioritization using EDT. Our previous work established that design thinking or human-centered design has been increasingly used in health research, especially as a participatory means of working with community members on health research [34]; however, it may not always be compatible with public health procedures and norms [35].

Focusing on equity while incorporating design into public health and PCOR may provide a more appropriate approach to address health equity [29,36]. In this study, these methods resulted in authentically engaged partnerships and researcher-participant trust, facilitated nonhierarchical conversations across and among people from different backgrounds, and fostered candid dialogue around key health and social issues affecting members from a community that has been impacted by structural racism [37]. Health equity approaches that focus on structural racism provide a tangible, workable, and viable strategy for increasing health equity and enhancing the overall population health [1].

Few of the burgeoning efforts to incorporate design into health research [38] specifically address equity [39], and even fewer directly address how structural racism or health disparities may need to be conceptualized in the light of design approaches. A recent call for action has been aimed at encouraging the use of equity-focused human-centered design to address behavioral and mental health [40].

Using an equity-based approach, this study sought to transform the research experience of the participants from a transactional to a meaningful engagement [41]. The participants were equipped with EDT skills and used them for creative problem solving to identify solutions to a pressing health issue, COVID-19. The activities broke down the roles of systems and power within the research setting as a means of directly addressing social injustice and the hierarchical power structure, which is the hallmark of health disparities [42]. The participants learned about the power of their own lived experiences to create and innovate research agendas related to PCOR and were able to ideate practical steps toward making changes through prototyping solutions for research priority setting, which has been identified as an effective form of engagement [5].

The free availability of the toolkit for download ensures that patient engagement researchers and community members can use and implement similar strategies. Future projects include expanding the toolkit to address other health issues severely impacting minoritized communities, such as perinatal mortality, and to provide a broader array of research environments for patient engagement.

### Limitations

The limitations of this study include the challenges in replicating the specific personnel and resources used, including technological resources and capacity. There were some technological challenges that affected the full participation of all stakeholders in the workshop activities. Some stakeholders did not have access to reliable or stable Wi-Fi services, which necessitated adjustments. Workshop activities relied on video and screen sharing; however, the stakeholders sometimes had to join the workshop via telephone and were only able to listen in. Some stakeholders dialed in on a mobile phone while using iPads (Apple Inc) to view the screen via a Zoom link. In addition, several stakeholders contracted COVID-19 during the project period, marking challenges in attending and completing the workshop activities. These reflect real challenges when engaging community members as partners in research. In addition, inviting stakeholders through referrals may have resulted in individuals being more likely to respond positively to and participate more actively in the activities than would be the case when implementing these activities in community settings; however, social desirability bias is widespread in all study designs.

### Conclusions

This study evaluated EDT strategies for community engagement in health research agenda setting, which provide an alternative to traditional top-down models and foster inclusive approaches. Despite the increasing use of human-centered design in health, only a few projects explicitly include equity in design thinking approaches. The use of methods and tools to intentionally engage underrepresented stakeholders in the process of research agenda setting and equitably sharing power between researchers and community members may improve health research, ultimately improving health equity.

## Abbreviations

CER: comparative effectiveness research
COREQ: Consolidated Criteria for Reporting Qualitative Studies
EDT: equity-centered design thinking
GRID: Grounding Health Research in Design
PCOR: patient-centered outcomes research

## Data Availability

The data sets generated and analyzed during this study are available from the corresponding author upon reasonable request.

## References

[1] Bailey ZD, Krieger N, Agénor M, Graves J, Linos N, Bassett MT (2017). Structural racism and health inequities in the USA: evidence and interventions. Lancet.

[2] Owen Jr WF, Carmona R, Pomeroy C (2020). Failing another national stress test on health disparities. JAMA.

[3] Yancy CW (2020). COVID-19 and African Americans. JAMA.

[4] Yancey AK, Ortega AN, Kumanyika SK (2006). Effective recruitment and retention of minority research participants. Annu Rev Public Health.

[5] Haynes SC, Rudov L, Nauman E, Hendryx L, Angove RS, Carton T (2018). Engaging stakeholders to develop a patient-centered research agenda: lessons learned from the research action for health network (REACHnet). Med Care.

[6] (2018). How PCORI Defines Stakeholders. Patient-Centered Outcomes Research Institute.

[7] (2013). Patient Centered Outcomes Research. Patient-Centered Outcomes Research Institute.

[8] (2021). Comparative Clinical Effectiveness Research. Patient-Centered Outcomes Research Institute.

[9] Olson M, Cottoms N, Sullivan G (2015). Engaging underrepresented minorities in research: our vision for a "Research-Friendly Community". Prog Community Health Partnersh.

[10] Chen E, Leos C, Kowitt SD, Moracco KE (2020). Enhancing community-based participatory research through human-centered design strategies. Health Promot Pract.

[11] Brown T, Wyatt J (2009). Design thinking for social innovation. Stanf Soc Innov Rev.

[12] Abookire S, Plover C, Frasso R, Ku B (2020). Health design thinking: an innovative approach in public health to defining problems and finding solutions. Front Public Health.

[13] Kolko J (2015). Design thinking comes of age. Harvard Business Review.

[14] Noel L (2016). Promoting an emancipatory research paradigm in Design Education and Practice. Proceedings of DRS 2016 International Conference: Future–Focused Thinking.

[15] An Introduction to Equity Centered Design. Intentional futures.

[16] Creative Reaction Lab.

[17] Gilmore-Bykovskyi A, Croff R, Glover CM, Jackson JD, Resendez J, Perez A, Zuelsdorff M, Green-Harris G, Manly JJ (2022). Traversing the aging research and health equity divide: toward intersectional frameworks of research justice and participation. Gerontologist.

[18] Reno R, Warming E, Zaugg C, Marx K, Pies C (2021). Lessons learned from implementing a place-based, racial justice-centered approach to health equity. Matern Child Health J.

[19] (2020). Coronavirus Disease 2019 Cases in the U.S. Centers for Disease Control and Prevention.

[20] (2021). QuickFacts: New Orleans, Louisiana. United States Census Bureau.

[21] (2013). New Orleans Community Health Improvement Report: Community Health Profile and Community Health Improvement Plan. New Orleans Health Department.

[22] (2022). Multicultural Guidelines for Communication Across Difference. VISIONS Inc.

[23] (2022). VISIONS Inc.

[24] Patton MQ (2010). Developmental Evaluation: Applying Complexity Concepts to Enhance Innovation and Use.

[25] Braun V, Clarke V (2006). Using thematic analysis in psychology. Qual Res Psychol.

[26] Braun V, Clarke V, Hayfield N, Terry G, Liamputtong P (2019). Thematic analysis. Handbook of Research Methods in Health Social Sciences.

[27] Tong A, Sainsbury P, Craig J (2007). Consolidated criteria for reporting qualitative research (COREQ): a 32-item checklist for interviews and focus groups. Int J Qual Health Care.

[28] PCORnet: A Network of Networks.

[29] Liburd LC, Hall JE, Mpofu JJ, Williams SM, Bouye K, Penman-Aguilar A (2020). Addressing health equity in public health practice: frameworks, promising strategies, and measurement considerations. Annu Rev Public Health.

[30] Milner A, Franz B, Henry Braddock J (2020). We need to talk about racism-in all of its forms-to understand COVID-19 disparities. Health Equity.

[31] Price-Haywood EG, Burton J, Fort D, Seoane L (2020). Hospitalization and mortality among Black patients and White patients with Covid-19. N Engl J Med.

[32] Sofolahan-Oladeinde Y, Mullins CD, Baquet CR (2015). Using community-based participatory research in patient-centered outcomes research to address health disparities in under-represented communities. J Comp Eff Res.

[33] Hasnain-Wynia R, Beal AC (2014). Role of the patient-centered outcomes research institute in addressing disparities and engaging patients in clinical research. Clin Ther.

[34] Bazzano AN, Martin J, Hicks E, Faughnan M, Murphy L (2017). Human-centred design in global health: a scoping review of applications and contexts. PLoS One.

[35] Bazzano AN, Martin J (2017). Designing public health: synergy and discord. Design J.

[36] Wallerstein N, Oetzel JG, Sanchez-Youngman S, Boursaw B, Dickson E, Kastelic S, Koegel P, Lucero JE, Magarati M, Ortiz K, Parker M, Peña J, Richmond A, Duran B (2020). Engage for Equity: a long-term study of community-based participatory research and community-engaged research practices and outcomes. Health Educ Behav.

[37] Paradies Y, Ben J, Denson N, Elias A, Priest N, Pieterse A, Gupta A, Kelaher M, Gee G (2015). Racism as a determinant of health: a systematic review and meta-analysis. PLoS One.

[38] Altman M, Huang TT, Breland JY (2018). Design thinking in health care. Prev Chronic Dis.

[39] Nijagal MA, Patel D, Lyles C, Liao J, Chehab L, Williams S, Sammann A (2021). Using human centered design to identify opportunities for reducing inequities in perinatal care. BMC Health Serv Res.

[40] Stiles-Shields C, Cummings C, Montague E, Plevinsky JM, Psihogios AM, Williams KD (2022). A call to action: using and extending human-centered design methodologies to improve mental and behavioral health equity. Front Digit Health.

[41] Forsythe L, Heckert A, Margolis MK, Schrandt S, Frank L (2018). Methods and impact of engagement in research, from theory to practice and back again: early findings from the Patient-Centered Outcomes Research Institute. Qual Life Res.

[42] Braveman PA, Kumanyika S, Fielding J, Laveist T, Borrell LN, Manderscheid R, Troutman A (2011). Health disparities and health equity: the issue is justice. Am J Public Health.

