# Correction: Improving the Engagement of Underrepresented People in Health Research Through Equity-Centered Design Thinking: Qualitative Study and Process Evaluation for the Development of the Grounding Health Research in Design Toolkit

**DOI:** 10.2196/58397

**Published:** 2024-04-04

**Authors:** Alessandra N Bazzano, Lesley-Ann Noel, Tejal Patel, C Chantel Dominique, Catherine Haywood, Shenitta Moore, Andrea Mantsios, Patricia A Davis

**Affiliations:** 1 Department of Social, Behavioral, and Population Sciences Tulane University School of Public Health and Tropical Medicine New Orleans, LA United States; 2 College of Design North Carolina State University Raleigh, NC United States; 3 Global Impact Board New Orleans, LA United States; 4 Louisiana Community Health Outreach Network New Orleans, LA United States; 5 Louisiana State University Health Sciences Center New Orleans, LA United States; 6 Public Health Innovation & Action New York, NY United States

In “Improving the Engagement of Underrepresented People in Health Research Through Equity-Centered Design Thinking: Qualitative Study and Process Evaluation for the Development of the Grounding Health Research in Design Toolkit” (JMIR Form Res 2023;7:e43101) the authors noted one error.

In Reference 28, the URL:

https://pcornet.org/about/

has been changed to:

https://www.pcori.org/engagement/engagement-resources/grid-toolkit

The correction will appear in the online version of the paper on the JMIR Publications website on April 4, 2024 together with the publication of this correction notice. Because this was made after submission to PubMed, PubMed Central, and other full-text repositories, the corrected article has also been resubmitted to those repositories.

